# Severe malaria in Europe: an 8-year multi-centre observational study

**DOI:** 10.1186/s12936-016-1673-z

**Published:** 2017-01-31

**Authors:** Florian Kurth, Michel Develoux, Matthieu Mechain, Denis Malvy, Jan Clerinx, Spinello Antinori, Ida E. Gjørup, Joaquím Gascon, Kristine Mørch, Emanuele Nicastri, Michael Ramharter, Alessandro Bartoloni, Leo Visser, Thierry Rolling, Philipp Zanger, Guido Calleri, Joaquín Salas-Coronas, Henrik Nielsen, Gudrun Just-Nübling, Andreas Neumayr, Anna Hachfeld, Matthias L. Schmid, Pietro Antonini, Tilman Lingscheid, Peter Kern, Annette Kapaun, José Saraiva da Cunha, Peter Pongratz, Antoni Soriano-Arandes, Mirjam Schunk, Norbert Suttorp, Christoph Hatz, Thomas Zoller

**Affiliations:** 1 Medizinische Klinik mit Schwerpunkt Infektiologie und Pneumologie, Charité Universitätsmedizin Berlin, Berlin, Germany; 2 Hôpital Saint-Antoine, Paris, France; 3 Section Tropical Medicine and Clinical International Health, Division of Infectious and Tropical Diseases, Department of Medicine, University Hospital Centre, Bordeaux, France; 4 Institute of Tropical Medicine, Antwerp, Belgium; 5 Department of Biomedical and Clinical Sciences L.Sacco, University of Milano, Milan, Italy; 6 Infectious Diseases Unit, Herlev University Hospital, Copenhagen, Denmark; 7 ISGlobal, Barcelona Center for International Health Research. (CRESIB), Hospital Clínic-Universitat de Barcelona, Barcelona, Spain; 8 Department of Medicine, National Centre for Tropical Infectious Diseases, Haukeland University Hospital, Bergen, Norway; 9 Department of Clinical Science, University of Bergen, Bergen, Norway; 10 National Institute of Infectious Diseases Lazzaro Spallanzani, Rome, Italy; 11 Department of Medicine I, Division of Infectious Diseases and Tropical Medicine, Medical University of Vienna, Vienna, Austria; 12 Institut für Tropenmedizin, Universität Tübingen, Tübingen, Germany; 13 SOD Malattie Infettive e Tropicali, Azienda Ospedaliero Universitaria Careggi, Florence, Italy; 14 Department of Infectious Diseases, Leiden University Medical Centre, Leiden, The Netherlands; 15 Section Tropical Medicine, Department of Internal Medicine I, University Medical Center Hamburg-Eppendorf, Hamburg, Germany; 16 Department of Clinical Research, Bernhard Nocht Institute for Tropical Medicine, Hamburg, Germany; 17 Institute of Public Health, University of Heidelberg, Heidelberg, Germany; 18 Travel Medicine Unit, Department of Infectious Diseases, Amedeo di Savoia Hospital-ASLTO2, Turin, Italy; 19 Tropical Medicine Unit, Hospital de Poniente, El Ejido, Spain; 20 Department of Infectious Diseases, Aalborg University Hospital, Aalborg, Denmark; 21 Department of Internal Medicine II, Section Infectious Diseases and Tropical Medicine, University Hospital Frankfurt/Main, Frankfurt/Main, Germany; 22 Swiss Tropical and Public Health Institute, Basel, Switzerland; 23 University of Basel, Basel, Switzerland; 24 Department of Infectious Diseases, Bern University Hospital and University of Bern, Bern, Switzerland; 25 Department of Infection & Tropical Medicine, Royal Victoria Infirmary, Newcastle upon Tyne, UK; 26 Clinica Luganese, Lugano, Switzerland; 27 Comprehensive Infectious Diseases Center, Department of Internal Medicine III, Ulm University Hospital, Ulm, Germany; 28 Section Clinical Tropical Medicine, Department of Infectious Diseases, University Hospital Heidelberg, Heidelberg, Germany; 29 Centro Hospitalar e Universitário de Coimbra, Coimbra, Portugal; 30 Division of Tropical Medicine and Infectious Diseases, Center of Internal Medicine II, University of Rostock, Rostock, Germany; 31 Hospital Universitari Joan XXIII, Tarragona, Spain; 32 Hospital Universitari Vall d’Hebron, Barcelona, Spain; 33 Division of Infectious Diseases and Tropical Medicine, Medical Center of the University of Munich (LMU), Munich, Germany

**Keywords:** Malaria, Falciparum, Severe malaria, Artesunate, Quinine, *Plasmodium*, Europe, Clinical study

## Abstract

**Background:**

Malaria remains one of the most serious infections for travellers to tropical countries. Due to the lack of harmonized guidelines a large variety of treatment regimens is used in Europe to treat severe malaria.

**Methods:**

The European Network for Tropical Medicine and Travel Health (TropNet) conducted an 8-year, multicentre, observational study to analyse epidemiology, treatment practices and outcomes of severe malaria in its member sites across Europe. Physicians at participating TropNet centres were asked to report pseudonymized retrospective data from all patients treated at their centre for microscopically confirmed severe *Plasmodium falciparum* malaria according to the 2006 WHO criteria.

**Results:**

From 2006 to 2014 a total of 185 patients with severe malaria treated in 12 European countries were included. Three patients died, resulting in a 28-day survival rate of 98.4%. The majority of infections were acquired in West Africa (109/185, 59%). The proportion of patients treated with intravenous artesunate increased from 27% in 2006 to 60% in 2013. Altogether, 56 different combinations of intravenous and oral drugs were used across 28 study centres. The risk of acute renal failure (36 vs 17% p = 0.04) or cerebral malaria (54 vs 20%, p = 0.001) was significantly higher in patients ≥60 years than in younger patients. Respiratory distress with the need for mechanical ventilation was significantly associated with the risk of death in the study population (13 vs 0%, p = 0.001). Post-artemisinin delayed haemolysis was reported in 19/70 (27%) patients treated with intravenous artesunate.

**Conclusion:**

The majority of patients with severe malaria in this study were tourists or migrants acquiring the infection in West Africa. Intravenous artesunate is increasingly used for treatment of severe malaria in many European treatment centres and can be given safely to European patients with severe malaria. Patients treated with intravenous artesunate should be followed up to detect and manage late haemolytic events.

## Background

Around 5200 cases of malaria are imported to EU countries per year, of which up to 10% progress to severe malaria [1]. Because most patients with imported malaria are not semi-immune, progression to severe malaria is considerably more frequent in non-endemic than in endemic countries. Non-immune patients carry a substantial risk of suffering from complications of the infection itself or from complications associated with intensive care treatment.

The epidemiology of imported severe malaria is changing. In addition to tourist or business travellers to tropical regions, migrants visiting friends and relatives (VFR) in their previous home country are increasingly affected [2, 3]. This population is less likely to seek pre-travel advice and to take anti-malarial prophylaxis [4, 5]. Most migrants are not aware of the waning of semi-immunity against malaria when they travel to their home countries.

Guidelines to define and to treat severe malaria have undergone major changes in the past decade at international and national levels. Criteria for the definition of severe malaria were amended by the World Health Organization (WHO) in 2006, 2010 and 2015, particularly with regard to the definition of hyperparasitaemia [6], and numerous classification and treatment recommendations still exist across European countries. Another challenge is the transition from intravenous quinine to intravenous artesunate as first-line treatment for severe malaria. Despite its superior potential to save lives and shorten duration of hospital and intensive care unit (ICU) treatment [7–10], many difficulties with regard to registration, availability and quality of artesunate have to be overcome before it will be easily available and widely used outside of specialist referral centres for tropical medicine in Europe. The pathophysiology of late haemolytic reactions occurring 2–6 weeks after treatment is not fully understood and harmonized guidelines for follow-up care of patients receiving this drug in Europe need to be developed [11, 12].

Data on epidemiology and treatment of imported severe malaria across Europe, together with a ‘road map’ towards drug approval of intravenous artesunate for the treatment of severe malaria in non-endemic countries are needed to eventually improve and harmonize treatment recommendations. Data are however available only from national cohorts and case registries. The European Network for Tropical Medicine and Travel Health (TropNet) [13] conducted an 8-year, multi-centre, observational study to analyse epidemiology, treatment practices and outcome of severe malaria in its member sites across 12 European countries.

## Methods

This multicentre observational study was performed among member sites of TropNet. All patients with microscopically confirmed severe falciparum malaria, according to the 2006 WHO criteria treated at one of the participating TropNet centres between 2006 and 2014, were eligible. Physicians at participating TropNet centres were asked to report pseudonymized retrospective data on demographic characteristics, medical and travel history, clinical presentation, anti-malarial drug regimen, supportive treatment, parasitaemia before and under treatment, complications under treatment, adverse drug reactions, outcome and follow-up during 28 days from all patients treated for severe malaria at their centre. The treatment remained the responsibility of the treating physician. Parasite clearance time was defined as time to the first thick blood smear without evidence of asexual parasites after initiation of anti-malarial treatment. For the analysis of this dataset, post-artemisinin delayed haemolysis (PADH) was defined as a decrease in haemoglobin together with signs of haemolysis (elevated LDH) after completion of anti-malarial treatment and complete parasite clearance. Electronic case report forms were used for data entry and transmission to the coordinating centre at Charité University Hospital, Berlin, where data were transferred into a database and checked manually for plausibility.

The primary objective of this study was to assess clinical presentation, treatment and outcome in patients with imported severe malaria. Descriptive statistics was performed on sociodemographic, medical, treatment, and outcome data. Mann–Whitney U test (two groups, continuous data), Fisher exact test (two groups, categorical data), or Kruskal–Wallis test (>two groups continuous data) at a two-sided significance level of α = 0.05 were used for comparative analysis. Analysis of variance (ANOVA) for continuous data and Pearson Chi squared test for categorical data were used to test the distribution of demographical characteristics of patients over time. Data are displayed as median (±interquartile range). Statistical analysis was performed using JMP (JMP 7.0, SAS Institute Inc, NC, USA).

The study was approved by the Ethics Committee of Charité University Hospital, Berlin. Ethical clearance for transfer of retrospective pseudonymized patient data was sought at participating Tropnet centres according to local regulations.

## Results

From 2006 to 2014, 190 patients with severe falciparum malaria were reported by the participating 28 TropNet centres from 12 European countries. After excluding three cases without documented criteria for severe malaria and two cases with double reporting, 185 datasets were available for analysis. The number of patients per centre ranged from one to 31 patients. The number of reported patients per country is shown in Table 1.

**Table 1:** Number of study centres and reported cases per country (n = 185)

Country	Number of centres	Number of reported cases	% of total number of cases
Austria	2	7	4
Belgium	1	19	10
Denmark	2	14	7
France	2	52	28
Germany	7	15	8
Italy	5	50	27
Netherlands	1	4	2
Norway	1	7	4
Portugal	1	1	1
Spain	2	12	6
Switzerland	3	3	2
UK	1	1	1

### Demography, place of infection and anti-malarial prophylaxis

**Fig. 1:**
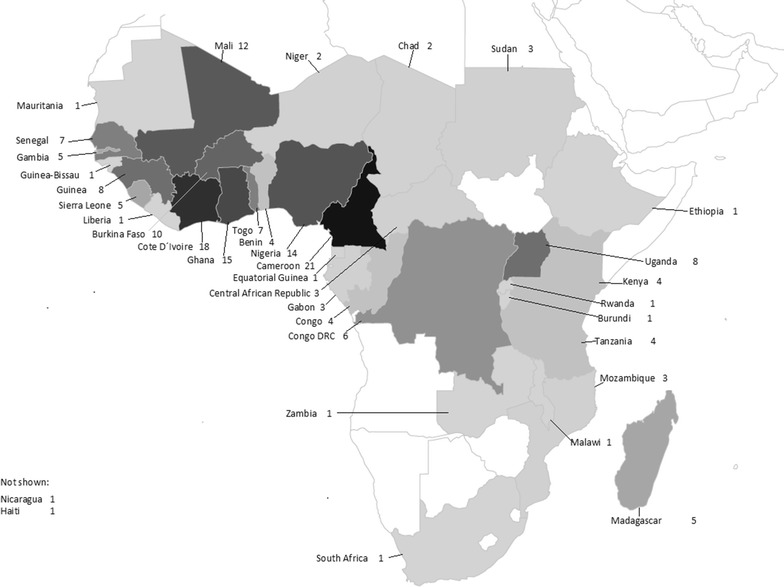
Countries where malaria infections where acquired (n = 185). Countries and number of cases per country are shown. *Gray*-*scale colour* corresponds to the proportion of cases acquired in the respective country

**Fig. 2:**
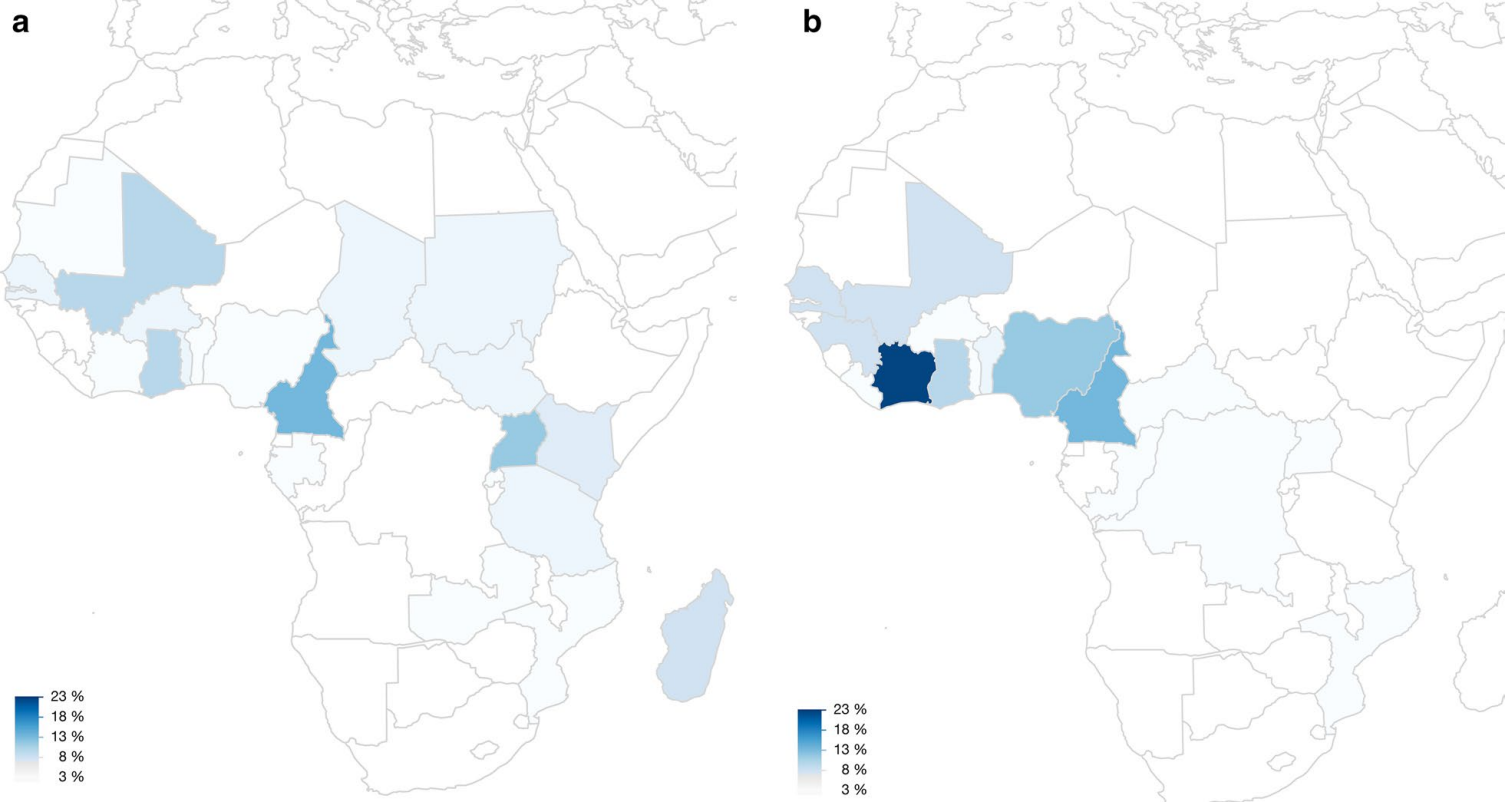
**a** Countries where European tourist travellers acquired malaria (n = 54). Colour intensity corresponds to the proportion of cases acquired in the respective country. **b** Countries where visiting friends and relatives travellers acquired malaria (n = 55). Colour intensity corresponds to the proportion of cases acquired in the respective country

Demographic data are shown in Table 2. The proportion of female patients was comparatively small (29%). The majority of patients (106/185, 57%) were of European origin without history of migration. Tourism was the main purpose of travel in Europeans (54/106, 51%), whereas VFRs were the predominant purposes of travel in patients with history of migration (55/68, 81%). European patients were on average older than patients with history of migration [median age 47 (IQR 33–57) vs 36 (IQR 27–45) years, p < 0.0001]. All malaria infections were acquired in Africa with the exception of two cases from Central America. By far the largest proportion of infections came from West Africa (109/185, 59%), followed by Central Africa (40/185, 22%), where Cameroon was the country with the highest number of imported cases (21/185, 11%, Fig. 1). VFR patients acquired malaria infections almost exclusively in West Africa, whereas European tourists acquired infections also in the tourist destinations of East Africa (Fig. 2). There was no change in age (F = 0.84, p = 0.5), gender (p = 0.11), origin of patients (p = 0.54) or purpose of travel (p = 0.10) during the 8-year course of the study. Almost 9 out of 10 patients (162/185, 88%) had not taken any anti-malarial chemoprophylaxis. Among 23 patients who took anti-malarial chemoprophylaxis, only six fully adhered to the prescribed regimen.

**Table 2:** Characteristics of patients with severe malaria (n = 185 patients in all categories)

Characteristics	n (median)	% [IQR]
Gender		
Male	132	71
Age		
Age in years	(42)	[31–52]
≥60 years	22	12
≤18 years	10	5
Origin of patients		
European, no history of migration	106	57
Immigrant/history of migration	68	37
Visitor from endemic country	11	6
Anti-malarial chemoprophylaxis		
None	162	88
Non-adherence to prescribed regimen	17	9
Doxycycline^a^	2	1
Chloroquine-proguanil^a,b^	2	1
Mefloquine^a^	1	1
Atovaquone-proguanil^a^	1	1
Criteria leading to classification as severe malaria		
Hyperparasitaemia >5%	132	71
Hyperparasitaemia >10%^c^	55	30
Hyperparasitaemia >2%^c^	154	83
Jaundice^d^	81	44
Impaired consciousness/coma	46	25
Acute renal failure	36	19
Liver function test >3 times upper normal	36	19
Circulatory collapse/shock	27	15
Anaemia <8 g/dl	27	15
Respiratory failure	22	12
Spontaneous/abnormal bleeding	13	7
Acidosis	9	5
Hypoglycaemia <40 mg/dl	6	3
Multiple convulsions	3	2
Number of criteria for severe malaria met by individual patients		
1	59	32
2	60	32
3	30	16
4	19	10
5	10	5
>5	7	3
Underlying co-morbidities		
Any	63	43
Hypertension	16	9
HIV	13	7
Diabetes	10	5
Chronic heart disease	8	4
Hepatitis	3	2
COPD	2	1
Other chronic conditions	11	6

Data are number of patients, unless indicated otherwise

*COPD* chronic obstructive pulmonary disease, *HIV* Human immunodeficiency virus

^a^ Patients with reported adherence to chemoprophylaxis only

^b^ Chloroquine-proguanil was taken for travel to Togo in 2007 and Burkina Faso in 2010

^c^ Hyperparasitaemia >2 and >10% were not used as criteria for severe malaria in this study according to WHO guidelines 2006 and are shown for informational purposes only

^d^ Clinical jaundice was used as criterion for severe malaria in this study according to WHO guidelines 2006

### Clinical presentation

Clinical manifestations and laboratory findings leading to classification as severe malaria are shown in Table 2. Median baseline parasitaemia was 6.5% (IQR 4–11) and hyperparasitaemia (≥5%) was the most common criterion of severe disease, followed by jaundice, which was a criterion for severe malaria in this study according to WHO guidelines as of 2006. Eight patients with jaundice had no other vital organ dysfunction and would not have been classified as severe disease according to WHO guidelines as of 2010 [14].

Underlying chronic conditions were found in 43% of patients (63/185), of which hypertension was the most frequent (9%, 16/185). Seven per cent of patients (13/185) were HIV positive. The majority of patients (119/185, 64%) met one or two criteria of severe malaria, whereas 8% (17/185) met more than four criteria. Patients ≥60 years presented, on average, with more criteria for severe malaria than younger patients (median 3 vs 2, *p* = 0.02). Table 3 shows the risk of presenting with a particular criterion of severe malaria according to age (≥60 years vs younger patients). There was no difference in type and number of criteria for severe malaria among patients of European versus non-European origin with the exception of a lower median baseline parasitaemia (7 vs 5%, respectively, *p* = 0.04).

**Table 3:** Risk of presenting with particular criteria of severe malaria according to age ≥60 versus <60 years

	Patients ≥60 years n = 22	Patients <60 years n = 163	p value
Cerebral malaria	12 (54)	34 (21)	0.001
Acute renal failure	8 (36)	28 (17)	0.04
Hyperparasitaemia	17 (77)	120 (73)	0.8
Jaundice	8 (36)	73 (44)	0.5
Liver function test >3 times upper normal	7 (31)	29 (17)	0.15
Shock	6 (27)	21 (13)	0.1
Anaemia	3 (14)	24 (15)	1.0
Respiratory failure	3 (14)	19 (12)	0.7
Acidosis	2 (9)	7 (4)	0.29
Spontaneous bleeding	3 (13)	10 (6)	0.19
Hypoglycaemia	1 (5)	5 (3)	0.53
Multiple convulsions	0 (0)	3(2)	1.0

Data are number of patients (%)

### Anti-malarial treatment

Intravenous quinine was the main first-line treatment in 93/185 patients (50%) whereas intravenous artesunate was used in 63/185 patients (35%). Seven patients (4%) received intravenous quinine and artesunate in combination. Table 4 gives an overview of the drugs and drug combinations used as follow-on treatment after intravenous therapy. Altogether 56 different combinations of intravenous and oral drugs were used across the different centres.

**Table 4:** Initial drug combinations and follow-on treatment in patients treated with intravenous quinine or intravenous artesunate for severe malaria

Patients	n	%
Treated with intravenous quinine	93	
Initial therapy		
Monotherapy	54	58
Combination with doxycycline	33	36
Combination with clindamycin	5	5
Combination with mefloquine	1	1
Follow-on treatment		
Oral quinine	29	31
Oral ACT	19	20
Oral AP	11	12
None^a^	34	37
Treated with intravenous artesunate	63	
Initial therapy		
Monotherapy	42	67
Combination with doxycycline	13	20
Combination with clindamycin	6	10
Combination with mefloquine	2/	3
Follow-on treatment		
Oral ACT	38	60
Oral AP	17	27
Oral quinine	2	3
Mefloquine	3	5
None^b^	3	5

Data are number of patients and %

*ACT* artemisinin-based combination therapy, *AP* atovaquone-proguanil

^a^ Quinine, doxycycline or clindamycin were given for at least 7 days, n = 2 patients died before initiation of subsequent therapy

^b^ Artesunate, doxycycline, clindamycin were given for at least 7 days

The proportion of patients treated with intravenous artesunate increased steadily during the course of the study from 27% (8/30) in 2006 to 60% (18/30) in 2013. In 22/185 patients (12%) only oral first-line treatments were given such as oral quinine (n = 16, exclusively in Italy), oral atovaquone-proguanil (n = 1, in a patient with HIV), and oral artemether–lumefantrine (n = 5). Patients treated with oral anti-malarials presented exclusively with hyperparasitaemia (n = 19, median parasitaemia 7%, range 5–8%) and/or jaundice (n = 6) as criteria for severe malaria and had no co-morbidities (except the one patient with HIV).

Choice of the first-line treatment was very heterogeneous across different sites and was mainly dependent on the country where the patient was treated. Centres in Norway, The Netherlands and Belgium reported treatment almost exclusively with intravenous artesunate, whereas the participating treatment centres in Spain and France used intravenous quinine in the majority of reported cases (9/12, 75% and 44/50, 88%, respectively).

### Concomitant and supportive treatment

An overview of supportive treatments is given in Table 5. Antibiotic therapy (44% of patients) and erythrocyte transfusion (21% of patients) were the most common. Erythrocyte exchange transfusion was performed in 8/185 (4%) patients at seven centres in Italy, Spain, Belgium, and The Netherlands. Seven of these patients were treated with intravenous quinine and one with intravenous artesunate. Baseline parasitaemia was >10% in all these patients (median 18%, IQR 10–27). Erythrocyte apheresis was performed in 5/185 patients (3%) at two centres (Vienna, Austria and Leiden, The Netherlands). Parasitaemia was >15% in all these patients (median 19%, IQR 18–28) and all five patients were treated with intravenous artesunate.

**Table 5:** Supportive treatments used in European patients with severe malaria (n = 185)

Supportive treatments	n	%
Antibiotic therapy	82	44
Erythrocyte transfusion	38	21
Vasopressor therapy	24	13
Mechanical ventilation	24	13
Invasive ventilation	18	
Non-invasive ventilation	6	
Haemodialysis and haemofiltration	20	11
Erythrocyte exchange transfusion	8	4
Erythrocyte apheresis	5	3

### Adverse drug reactions

Adverse drug reactions were reported in 27/100 patients (27%) treated with intravenous quinine, and in 21/70 (30%) patients treated with intravenous artesunate. None of them was fatal. Cinchonism was the most common adverse drug reaction in patients treated with intravenous quinine (19/100, 19%). It was rated as mild in 17/19 cases and moderate in 2/19 cases by the treating physician. Hypoglycaemia occurred in 4/100 patients (4%) and cardiac arrhythmias in 1/100 patient (1%) treated with intravenous quinine.

In patients treated with intravenous artesunate, PADH was reported in 19/70 patients (27%), a finding which first became known during the study period in the year 2011. Onset of PADH was reported during days 10 to 14 (median 14) and median duration of haemolysis was reported to be 14 (IQR 8–18) days. Three patients (15%) with PADH received blood transfusions, with 2 patients (10%) re-hospitalized (for 3–5 days, respectively). In 1 patient, PADH was reported after therapy with only oral artemether–lumefantrine. This patient and some of the other patients with delayed PADH have already been reported elsewhere [15–17].

In one patient, an acute cerebellar syndrome (ataxia, dysarthria, dysmetria, adiadochokinesis) was described beginning 3 days after the end of anti-malarial treatment (day 10). The patient had not shown any neurological symptoms during the acute phase of malaria and had been treated with a loading dose of intravenous quinine on day 1, intravenous artesunate from day 1 to 4 and oral artemether–lumefantrine from day 5 to 7. An MRI scan and lumbar puncture showed no abnormalities. Due to persistence of symptoms, the patient received physiotherapy until ten weeks after anti-malarial treatment, where neurological symptoms steadily improved. Complete recovery was reported 7 months after the malaria episode.

### Outcome

Three patients died, two of European origin and one with history of migration, resulting in a 28-day survival rate of 98.4%. All three patients had been treated with intravenous quinine and one of them also with intravenous artesunate simultaneously. All deaths occurred within the first 3 days after admission. All three patients had presented with hyperparasitaemia (9, 10 and 40%) and respiratory distress requiring mechanical ventilation. Respiratory distress with the need for mechanical ventilation was significantly associated with the risk of death in the study population (13 vs 0%, p = 0.001). Of note, two of the patients who died were 22–34 years of age, respectively, and had no underlying co-morbidities. One of them had a history of migration. The third patient was 70 years of age, suffered from a pre-existing chronic cardiomyopathy and died from therapy-refractory shock.

In 76% of patients (117 of 153 patients with available data) treatment took place in an ICU, where the median length of stay was three (IQR 2–5) days. The median length of inpatient treatment was 7 days (IQR 5–9). Median time to 99% parasite clearance was 48 h (IQR 24–72, n = 126) and median time to complete parasite clearance, was 72 h (IQR 60–120, n = 104). Data showing shorter parasite clearance time and shorter ICU and inpatient treatment in patients treated with intravenous artesunate compared to intravenous quinine were reported elsewhere [8]. The 22 patients who received oral anti-malarial treatment had comparatively long median 99% parasite clearance and complete parasite clearance times (72 h, IQR 48–72, and 120 h, IQR 84–144, respectively). There were no documented early or late parasitological failures.

Among 46 patients with cerebral malaria, six had neurological sequelae at discharge such as confusion, dysphasia, ataxia, and imbalance. In four patients (2/25 treated with artesunate vs 2/21 treated with quinine) confusion and ataxia persisted at day 28. Among 36 patients with acute renal failure at presentation, 13 (36%) patients had elevated creatinine levels at discharge, which had not been reported by the patient or documented in medical charts before malarial infection. In nine of these patients (4/17 treated with artesunate vs 5/16 treated with quinine) elevated creatinine levels persisted after day 28. Two patients suffered from necroses of fingers and toes as sequelae of vasopressor therapy during malaria.

Among eight patients with jaundice as only criterion for severe malaria, who would not have been classified as severe according to WHO 2010 criteria, none experienced documented complications of treatment or sequelae, seven were not treated at an ICU and patients had a comparably short median length of stay in hospital of 4 days (IQR 4–6).

## Discussion

Imported malaria remains a relevant clinical problem due to the rapid potential progression to severe and life-threatening disease in non-immune patients. This study presents clinical and treatment data from 28 centres of the TropNet from 12 countries, one of the largest databases collected on this patient population to date. Previous studies on severe malaria in Europe are only available either at centre [18–20] or country [3, 21–23] level.

### Anti-malarial treatment—intravenous artesunate

Intravenous artesunate has been shown to improve survival in patients with severe malaria in endemic areas, with particular benefit for patients with high parasitaemia [>10% infected red blood cells (RBCs)] [7, 9, 10]. A randomized, controlled trial to confirm superiority of artesunate over quinine in non-endemic areas would be unethical. Other benefits of treatment with intravenous artesunate such as shorter ICU and hospital treatment were clearly demonstrated in European patients [8].

Quinine is still widely used in Europe, but the rate of patients receiving intravenous artesunate almost doubled in the 8-year study period. In the final year 2014, every second patient received intravenous artesunate. Current surveillance data from national reference centres indicate that the proportion of patients treated with artesunate is further increasing, particularly in countries where participating centres still reported frequent treatment with quinine during this study [24]. Although prospectively collected safety data from Europe is not available, this study adds to the evidence that artesunate is effective and can safely be used to treat patients in Europe. Artesunate is used in Europe despite considerable legal problems: The manufacturer of intravenous artesunate has been prequalified for good manufacturing practice-standard (GMP) by WHO [6], but the drug is not available in a European GMP-standard quality. It has an orphan designation for Europe by the European Medicines Agency since 2007, but no marketing authorization in Europe or in USA. Only in France, USA, Belgium, Denmark, and The Netherlands, named-patient programmes or similar protocols are in place, providing a legal basis for treating patients with this lifesaving drug [25, 26]. Until intravenous artesunate receives approval from the European Medicines Agency and the US Food and Drug Administration, the legal context for physicians procuring and applying this drug in Europe will remain unsatisfactory. This study also highlights the differences in treatment practices and guidelines across Europe: from occasional use in some countries to exclusive use of artesunate to treat severe malaria in The Netherlands or Norway. This finding likely reflects the respective national legal framework for using non-licensed drugs as well as national treatment recommendations [27]. The treatment of patients with intravenous artesunate and quinine in parallel, reported in seven patients, might also be based on judicial reasons: physicians might want to combine the most effective but unapproved drug (artesunate) with the approved and recommended standard first-line treatment (quinine) in order to avoid a legal risk [28]. No benefit of this combination has been shown compared to treatment with artesunate alone, whereas the frequency of adverse events increased [29]. A small number of patients were treated exclusively with oral anti-malarials in this study. These patients were less severely ill, including three patients with jaundice, as the only criterion for severe disease, who would not have been classified as severe according to the current WHO classification. No treatment complications were reported for these patients, yet time to parasite clearance was comparatively long. Overall, there was a remarkable variety of altogether 56 different combinations of intravenous and oral anti-malarial drugs reported. Harmonized, evidence-based European treatment guidelines would be useful to support clinicians in their choice of anti-malarial treatments.

### Adverse drug reactions

Following the initial description of an episode of severe prolonged haemolysis after treatment of a patient with severe malaria with intravenous artesunate in Japan in 2002 [30], late haemolytic reactions 2–6 weeks after treatment with intravenous artesunate were described in a case series in European patients in 2011 [12], and then confirmed in studies in European [15–17] and African [31] patients. Removal of parasites from RBCs in the spleen, leaving behind a once-infected ‘pitted’ erythrocyte with a shorter life span has been shown to be a potential mechanism causing late haemolysis [11], but the pathophysiology is not fully understood. The rate of haemolytic reactions and transfusions reported in the literature is variable depending on size, context, type, and setting of a study as well as on definitions of post-treatment haemolysis; the results of the present study are generally in line with previous observations [12, 16, 32] and late haemolysis can be expected to occur in approximately 20–30% of non-immune patients treated. As shown by the present data, a considerable proportion of them also receive blood transfusions. The results show once more that patients receiving intravenous artesunate for treatment of severe malaria should be routinely observed for signs of haemolysis at least on days 7 and 14 after treatment. An acute cerebellar syndrome 3 days after the end of anti-malarial treatment with intravenous artesunate and oral artemether lumefantrine was reported in one patient. Although the reported time until complete resolution (7 months) is longer than in most cases reported in the literature; symptoms and time of onset are suggestive of post-malaria neurological syndrome [33].

### Mortality

Mortality in this patient population was very low, reflecting the high standard of intensive care in Europe. Previous single-centre and national studies reported mortality rates between 4 and 15% [3, 18, 19, 22, 34]. The proportion of patients who had criteria of severe malaria associated with adverse outcome and death was comparable to other studies (Table 2) [2, 21, 34]. Mortality might have been biased by the fact that most reporting centres are tertiary care institutions with long experience in treating severe malaria. The study did not capture cases of severe malaria in smaller remote hospitals, where mortality might be higher. The increasing use of intravenous artesunate as main first-line treatment may also have contributed to reduce mortality, e.g., through rapid parasite clearance and shorter length of ICU and inpatient treatment [7–9]. Age as risk factor for adverse outcome of imported severe malaria has been shown by numerous studies [2]. In the present study, population patients ≥60 years were more likely to suffer from acute renal failure or from cerebral malaria, yet there was no increased case fatality among older patients.

### Anti-malarial chemoprophylaxis

Only 10% of patients with severe malaria had taken anti-malarial chemoprophylaxis and very few of them had been fully compliant. These data suggest that correct anti-malarial prophylaxis can effectively prevent severe malaria in European travellers. Counselling of travellers on malaria prevention should be improved and coverage increased, particularly for travellers going to West Africa, where 60% of infections in this study were acquired. Little is known about the proportion of European travellers who take prophylaxis. In a recent study only 60% of travellers from the UK to endemic areas used anti-malarial chemoprophylaxis [35]. VFR travellers are a large traveller population to Africa with different perceptions of malaria and its prevention [4, 5]. This may also influence the longer delay between onset of symptoms of malaria and presentation in hospital. The fact that 37% of patients in the present study had a history of migration clearly demonstrates the risk of these patients to suffer from severe malaria. Moreover, one of the patients who died was a 34 years old, otherwise healthy patient with history of migration. She presented with hyperparasitaemia, acute renal failure, jaundice, and respiratory failure. There was altogether no difference in symptoms and clinical presentation between patients with or without history of migration, suggesting a waning of semi-immunity in migrants who left endemic areas [36].

### Supportive treatments

Supportive treatments such as exchange transfusions and erythrocyte apheresis are a matter of controversy and their use is guided by national or local practices. European single-centre studies recently failed to demonstrate improved parasite clearance through whole blood or erythrocyte exchange, compared to patients treated with quinine or artesunate alone [37, 38]. Only 4% of patients received exchange transfusions in this study. It was performed in only 7 out of 28 participating centres, mainly in patients treated with intravenous quinine. Likewise, only five patients in two centres received automated erythrocyte apheresis. With the increased use of intravenous artesunate and its potential to rapidly reduce high parasite loads it needs to be determined whether particular patient populations might still benefit from these adjunctive treatments [39].

## Limitations

This observational study has inherent limitations. As patient information was collected retrospectively in TropNet centres and not all patients treated may have been reported equally, selective under-reporting, e.g., for patients who died, may have occurred. Bias in reporting data on PADH must be assumed as the condition was not known at the beginning of the study and no universally accepted clinical definition exists to date. All TropNet centres are referral centres for tropical medicine and patient composition as well as treatment data may not fully reflect treatment practices and outcomes in non-referral hospitals. Moreover, data reported by the participating treatment centres may not always fully reflect treatment practices in the respective countries. For patients with elevated creatinine levels at the end of follow-up, it cannot be ruled out that unknown or undocumented elevation of creatinine had existed before the episode of severe malaria.

## Conclusion

The data show that the majority of patients with severe malaria in Europe are tourists or migrants acquiring infection in West Africa. Intravenous artesunate is increasingly used for treatment of severe malaria; it is the most effective drug and can be safely given to European patients with severe malaria. There is need for harmonization of guidelines for the treatment of severe malaria in Europe. Patients treated with intravenous artesunate should be followed up to detect late haemolytic events.
